# Bibliometric analysis of research on pediatric hypothyroidism treatment: a global perspective on trends and emerging fronts (2018–2026)

**DOI:** 10.3389/fped.2026.1908940

**Published:** 2026-08-17

**Authors:** Wei Wu, Zhengyun Sun, Mengxun Liu, Yu Huang, Tingting Liu, Xiaobing Qiu, Yuanbiao Wang

**Affiliations:** 1 Children’s Health Section, Ganzhou Women and Children’s Health Care Hospital, Ganzhou, Jiangxi, China; 2 Department of Radiology, Lincang First People’s Hospital, Lincang, Yunnan, China; 3 Department of Colorectal Surgery, The Third Affiliated Hospital of Kunming Medical University/Yunnan Cancer Hospital, Kunming, Yunnan, China

**Keywords:** congenital hypothyroidism, levothyroxine, neonatal screening, pediatric hypothyroidism, subclinical hypothyroidism

## Abstract

**Background:**

Pediatric hypothyroidism is among the most prevalent endocrine disorders in childhood, yet no bibliometric analysis has systematically mapped the research landscape of its treatment.

**Objective:**

This study aimed to characterize the publication trends, geographic distribution, research hotspots, and emerging fronts in pediatric hypothyroidism treatment research from 2018 to 2026.

**Methods:**

A comprehensive search was conducted in the Web of Science Core Collection (WoSCC) as the primary database, with external data validation performed via PubMed. Bibliometric indicators were analyzed using CiteSpace, Python and R. Keyword co-occurrence networks were constructed with Louvain community detection, and burst detection was performed to identify emerging research fronts.

**Results:**

A total of 1,302 publications were included. Annual output grew from 138 (2018) to a peak of 183 (2022). The United States (*n* = 263, 20.2%), China (*n* = 203, 15.6%), and Italy (*n* = 137, 10.5%) were the leading contributors. Five thematic clusters were identified: (1) congenital hypothyroidism and neonatal screening; (2) subclinical hypothyroidism; (3) pregnancy-related thyroid disorders; (4) autoimmune thyroid disease; and (5) comorbidities and genetic syndromes. Burst detection revealed “obesity” (burst strength = 7.2), “COVID-19” (4.0), and “thyroid dyshormonogenesis” (3.2) as emerging fronts.

**Conclusions:**

The field is shifting focus from congenital hypothyroidism screening optimization toward subclinical hypothyroidism management and the recognition of obesity-related TSH elevation as a distinct physiological entity. Future research should prioritize prospective trials on watchful-waiting strategies and obesity-specific TSH reference ranges.

## 1 Introduction

Thyroid hormones are indispensable for normal brain maturation, linear growth, and metabolic homeostasis during childhood. Hypothyroidism in children, whether congenital or acquired, remains one of the most prevalent endocrine disorders worldwide (1). Congenital hypothyroidism (CH) is the most common neonatal endocrine condition. It affects approximately 1 in 2,000 to 1 in 4,000 newborns globally, with incidence rates varying by population and screening methodology (2). Severe neonatal hypothyroidism, if left untreated, causes irreversible neurodevelopmental impairment, including diminished IQ scores, motor deficits, and hearing dysfunction (3, 4). Subclinical hypothyroidism (SCH), defined as elevated serum thyroid-stimulating hormone (TSH) with normal free thyroxine (FT4) levels, is increasingly recognized in pediatric populations. Reported prevalence rates range from 3 to 15%, depending on the reference ranges employed (5). Thyroid hormone deficiency also impairs linear growth, pubertal development, bone mineral accrual, lipid metabolism, and cardiovascular function in growing children (6).

Levothyroxine (L-T4) has served as the cornerstone of thyroid hormone replacement therapy for over four decades. Its efficacy in preventing neurocognitive sequelae, when initiated promptly after neonatal screening, is well established (7). Current guidelines from the European Society for Paediatric Endocrinology (ESPE) and the American Thyroid Association (ATA) recommend L-T4 as the first-line agent. These guidelines recommend initial doses of 10–15 μg/kg/day for CH, with individualized titration guided by serial TSH monitoring (8). Nevertheless, significant clinical controversies persist. The optimal TSH threshold for initiating treatment in SCH remains debated. Some experts advocate intervention at TSH > 10 mIU/L, whereas others reserve treatment for TSH persistently above 7–8 mIU/L or for patients with concomitant risk factors such as obesity, autoimmune thyroiditis, or genetic syndromes (9). Vulnerable subpopulations, including children with Down syndrome, Prader-Willi syndrome, and Turner syndrome, present additional diagnostic and therapeutic challenges. These children often exhibit altered thyroid physiology and atypical clinical presentations, which increase the risk of both overdiagnosis and undertreatment (10). Emerging evidence further suggests that SARS-CoV-2 infection may transiently disrupt thyroid function in children. This finding raises questions about post-pandemic surveillance strategies (11).

Despite the growing volume of clinical trials, consensus guidelines, and narrative reviews, no study has systematically mapped the bibliometric landscape of pediatric hypothyroidism treatment research (12–14). Traditional systematic reviews and meta-analyses focus on specific clinical questions, such as optimal L-T4 dosing or the natural history of SCH (15). However, these approaches cannot capture the macro-level structure of the field, including the evolution of research themes, the geographic distribution of contributions, and the emergence of novel research fronts (16). The literature has expanded rapidly since 2018, driven by updated international guidelines, the proliferation of genomic studies on thyroid dyshormonogenesis, and post-pandemic reassessment of thyroid screening protocols (17). A comprehensive bibliometric synthesis is therefore both timely and warranted.

The present study addresses this gap through a systematic bibliometric analysis of pediatric hypothyroidism treatment research indexed in the Web of Science Core Collection (WoSCC) from 2018 to 2026. Specifically, we aimed to: (1) characterize temporal trends in publication output and citation impact; (2) identify the leading countries, institutions, and authors driving the field; (3) map the intellectual structure through keyword co-occurrence analysis and thematic clustering using network analysis and community detection algorithms; and (4) detect emerging research fronts and burst keywords that signal future directions. By providing a panoramic view of the research landscape, this study seeks to inform clinical researchers, guideline developers, and funding agencies about the current state of knowledge and the most pressing unanswered questions in pediatric hypothyroidism management.

## 2 Materials and methods

### 2.1 Data sources

The Web of Science Core Collection (WoSCC), comprising the Science Citation Index Expanded (SCIE) and Social Sciences Citation Index (SSCI), was selected as the data source for its comprehensive citation metadata and established use in bibliometric studies (18, 19). The integrated SCIE + SSCI database ensures inclusion of high-quality publications across both clinical and social science domains relevant to pediatric endocrinology. To mitigate potential selection bias and ensure the robustness of our bibliometric findings, PubMed was utilized as an external validation database. We extracted the annual publication records and the top 20 most frequent Medical Subject Headings (MeSH) and author keywords from the PubMed validation dataset to cross-reference with the core WoSCC trends and keyword clusters.

### 2.2 Search strategy

To ensure a comprehensive search, a combination of synonymous and closely related terms was utilized to construct search formulas. Articles were retrieved using the topic search function in WoSCC. The search formulas were comprehensively determined based on the target population, condition, and intervention, which can be detailed as follows: (1) Population: “pediatric” OR “paediatric” OR “child” OR “children” OR “childhood” OR “infant” OR “neonat” OR “newborn” OR “adolescent” OR “teenager” OR “juvenile” OR “preschool” OR “pubert” OR “youth”; (2) Condition: “hypothyroidism” OR “hypothyroid” OR “congenital hypothyroidism” OR “subclinical hypothyroidism” OR “primary hypothyroidism” OR “central hypothyroidism” OR “thyroid insufficiency” OR “Hashimoto” OR “autoimmune thyroiditis” OR “athyreosis” OR “thyroid dysgenesis” OR “hypothyroxinemia”; (3) Intervention: “treatment” OR “therapy” OR “management” OR “intervention” OR “levothyroxine” OR “L-thyroxine” OR “LT4” OR “thyroid hormone” OR “hormone replacement” OR “dosage” OR “screening” OR “newborn screening” OR “outcome” OR “follow-up” OR “euthyroid*”. The three blocks were combined using the Boolean operator AND. The search was restricted to articles and reviews published in English. No lower publication year limit was imposed during database retrieval to capture the full scope of the literature. During the preliminary screening process, studies published before 2018 were excluded to focus the analysis on contemporary research trends, and the final bibliometric analysis included publications from 2018 to 2026.

For external validation, the WoSCC search strategy was adapted to the PubMed/MEDLINE database using the same conceptual framework (population, condition, and intervention) with equivalent free-text keywords and PubMed-specific field tags [e.g., (Title/Abstract)]. The PubMed dataset (2018–2026) was subsequently used to validate the consistency of publication trends and research hotspots identified in the WoSCC analysis.

### 2.3 Inclusion and exclusion criteria

Articles were included if they: (1) were original articles or review articles indexed in the WoSCC; (2) addressed thyroid hormone deficiency or hypothyroidism in pediatric populations (aged 0–18 years); and (3) involved aspects of treatment, management, or therapeutic outcomes. Pregnancy-related studies were included only if they addressed neonatal or childhood thyroid outcomes or had direct relevance to pediatric hypothyroidism management. Articles were excluded if they: (1) focused exclusively on adult populations without pediatric subgroup data; (2) addressed hyperthyroidism or thyroid malignancy as the primary condition without relevance to hypothyroidism management; (3) were editorial materials, conference abstracts, letters, or retracted publications; or (4) were duplicate records.

### 2.4 Data extraction and processing

The literature search was finalized on May 20, 2026. Following application of the predefined eligibility criteria and the publication-year restriction (2018–2026), 1,310 records were retrieved from the WoSCC. After removing 8 duplicate records, a final dataset of 1,302 unique publications was retained for analysis. Metadata including publication year, authors, institutional affiliations, countries/regions, source journal, author keywords, Keywords Plus, abstract, and cited references were programmatically extracted using a custom Python script (version 3.9.13). Country and institutional information were parsed from the C1 (Author Address) field using regular expression pattern matching. Author names were standardized to the “Lastname, Initials” format to resolve inconsistencies across records**.**

### 2.5 Bibliometric analysis

After screening all eligible publications, R software (version 4.5.1) and CiteSpace (version 6.4.2) were used to analyze publication information on pediatric hypothyroidism treatment over the specified period. Each of these tools has its own strengths and can effectively complement each other in the process of conducting bibliometric analysis. CiteSpace (20) is a visual analysis and bibliometrics tool developed to visually map highly cited and pivotal documents and analyze the emergence of research topics (21). In this study, CiteSpace was primarily used to analyze the dual-map overlay of the journals and detect the citation bursts of keywords. R software, utilizing the bibliometrix package alongside ggplot2 and networkD3, provides a trend of publications by institutions, countries, and authors, as well as multidimensional scientific mapping (22). It was employed to construct the three-field Sankey diagram, keyword co-occurrence networks, and Louvain community detection (23, 24). This analysis encompassed various aspects including authors, countries, institutions, journal distribution, references, and keywords.

## 3 Results

### 3.1 Overview of the literature

**Figure 1:**
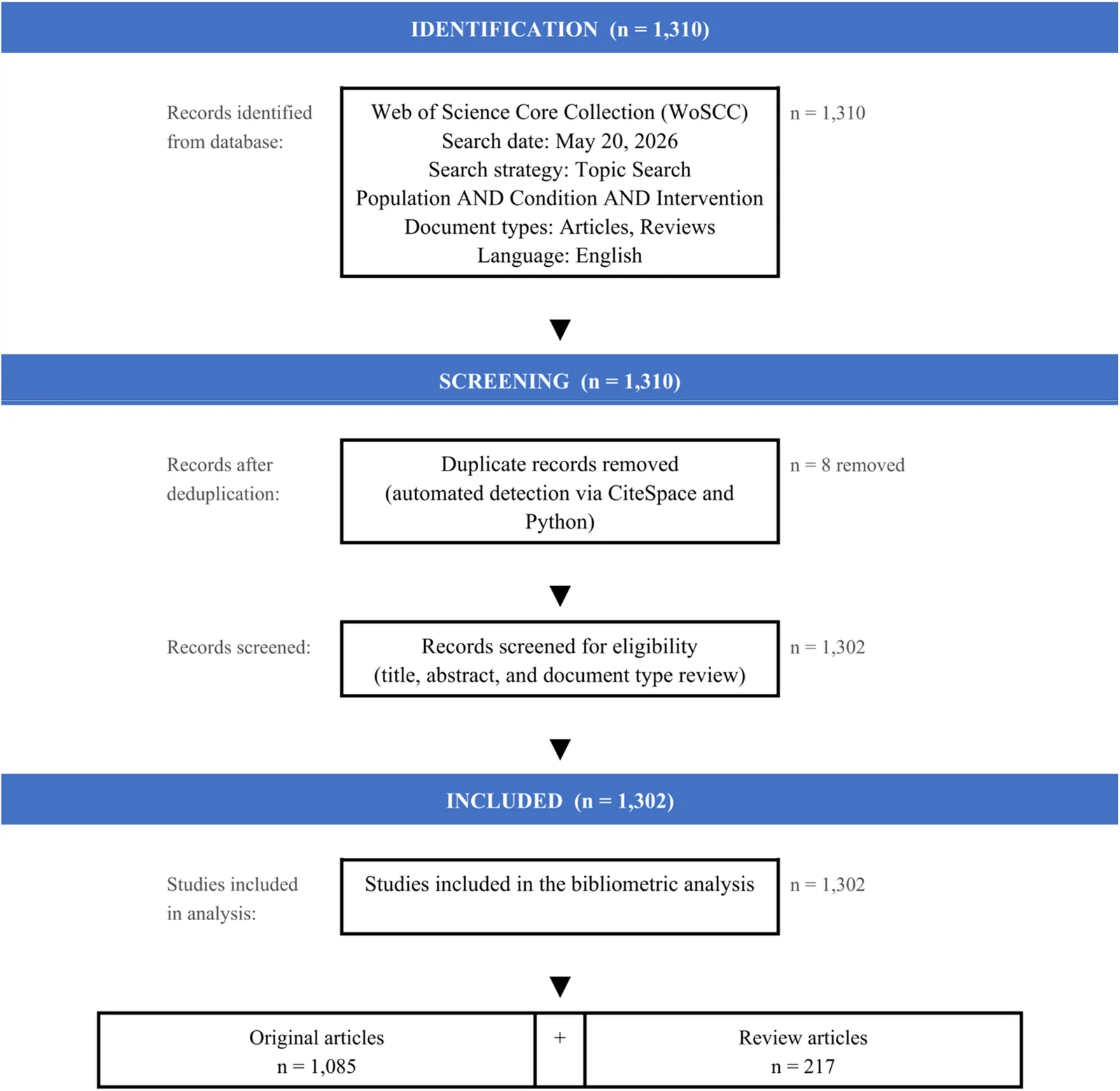
PRISMA flow diagram of the literature search and selection process. The numbers of identified, screened, excluded, and included records are shown. PRISMA, Preferred Reporting Items for Systematic Reviews and Meta-Analyses.

The selection process is summarized in Figure 1. In this study, a total of 1,302 publications were included in the final analysis, comprising 1,085 original articles and 217 review articles. As detailed in Table 1, which outlines the search strategy and inclusion/exclusion criteria, these publications were authored by researchers from 30 countries/regions and published across 454 distinct journals. The dataset contained 50 high-frequency author keywords. The keyword co-occurrence network comprised 25 nodes and 38 edges after keyword standardization and threshold filtering.

**Table 1:** Search strategy and inclusion/exclusion criteria.

Category	Search Terms (Boolean Logic)	Inclusion Criteria	Exclusion Criteria
Population (P)	pediatric OR paediatric OR child* OR infant* OR neonat* OR newborn* OR adolescent* OR teenager* OR juvenile OR preschool OR pubert*	Pediatric population aged 0–18 years	Adult-only studies without pediatric subgroup data
Condition (C)	hypothyroidism OR hypothyroid OR congenital hypothyroidism OR subclinical hypothyroidism OR primary hypothyroidism OR central hypothyroidism OR thyroid insufficiency OR Hashimoto OR autoimmune thyroiditis OR athyreosis OR thyroid dysgenesis OR hypothyroxinemia	Studies addressing thyroid hormone deficiency or hypothyroidism	Studies focusing exclusively on hyperthyroidism or thyroid malignancy
Intervention (I)	treatment OR therapy OR management OR intervention OR levothyroxine OR L-thyroxine OR LT4 OR thyroid hormone* OR hormone replacement OR dosage OR screening OR newborn screening OR outcome* OR follow-up OR euthyroid*	Studies involving treatment, management, or therapeutic outcomes	Conference abstracts, editorial materials, letters, or retracted publications
Combined	(P) AND (C) AND (I)	Articles and reviews in English, indexed in WoSCC	Duplicate records; non-English publications

### 3.2 Annual publication trends

**Figure 2:**
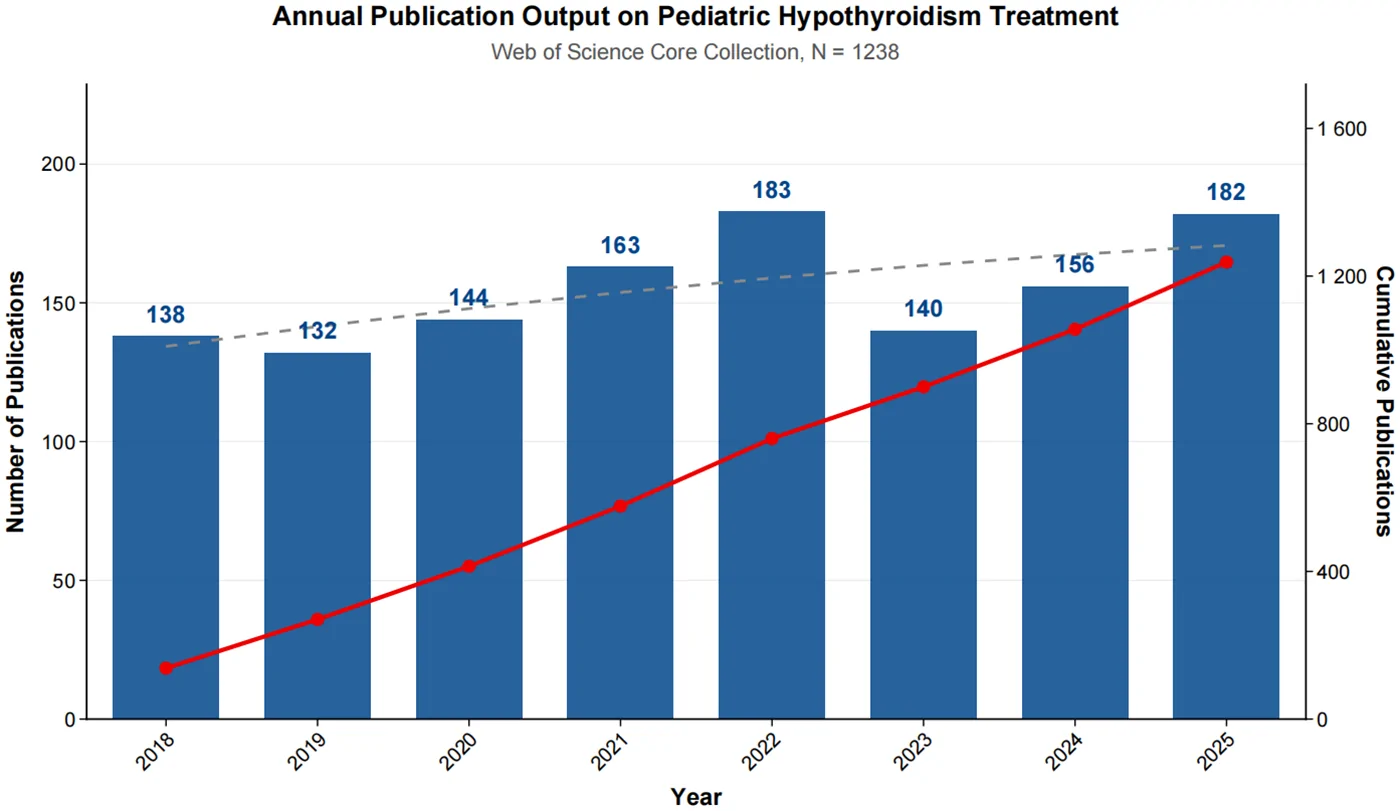
Annual publication trends in pediatric hypothyroidism treatment research from 2018 to 2026.

The annual publication volume and growth trends are illustrated in Figure 2. Publication output exhibited a generally upward trajectory over the study period. Output increased from 138 articles in 2018 to a peak of 183 articles in 2022, representing a 32.6% growth. A modest decline occurred in 2023 (*n* = 140), followed by recovery in 2024 (*n* = 156) and a secondary peak in 2025 (*n* = 182). By mid-2026, the cumulative number of publications had reached 1,302. A quadratic trend line fitted to the annual data indicates sustained growth momentum, with a mean annual output of approximately 155 publications. External validation using the PubMed database demonstrated consistent publication trends with the WoSCC dataset (Spearman's *ρ* = 0.90, *P* = 0.002; Supplementary Figure S1). In addition, comparison of MeSH terms and author keywords showed a 95% overlap in core research topics (Supplementary Table S1), supporting the reliability of the WoSCC dataset for subsequent analyses.

### 3.3 Geographic and institutional contributions

**Figure 3:**
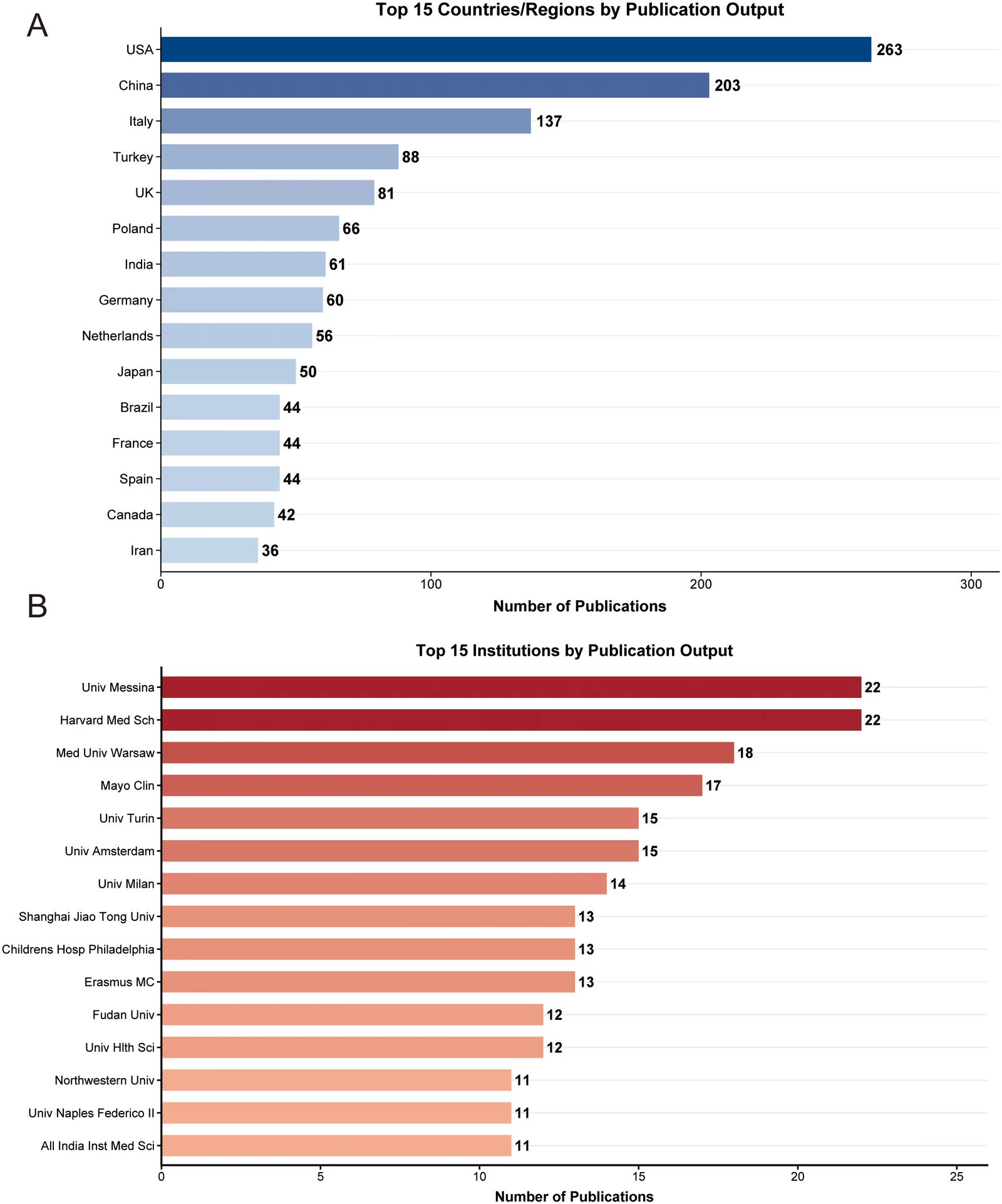
Geographic and institutional distribution of publications. **(A)** The top 15 most productive countries/regions. **(B)** The top 15 most productive institutions.

The geographic analysis revealed that the United States (*n* = 263, 20.2%) was the leading contributor to the literature, followed by China (*n* = 203, 15.6%), Italy (*n* = 137, 10.5%), Turkey (*n* = 88, 6.8%), and the United Kingdom (*n* = 81, 6.2%) (Figure 3A). Together, the top five countries accounted for 59.3% of all publications. The remaining top-10 contributors included Poland (*n* = 66), India (*n* = 61), Germany (*n* = 60), the Netherlands (*n* = 56), and Japan (*n* = 50).

At the institutional level, Figure 3B details the most prolific organizations. The University of Messina (Italy) and Harvard Medical School (United States) jointly held the leading position, contributing 22 publications each. They were followed by the Medical University of Warsaw (Poland, *n* = 18), Mayo Clinic (United States, *n* = 17), and the University of Turin (Italy, *n* = 15). It is worth mentioning that the presence of multiple Italian institutions among the top contributors profoundly reflects the country's strong and historical research tradition in pediatric endocrinology. Furthermore, notable contributions from the University of Amsterdam (*n* = 15), Erasmus MC (*n* = 13), and Shanghai Jiao Tong University (*n* = 13) further illustrate the extensive international scope of research activity. Table 2 comprehensively summarizes the exact publication metrics for these leading countries and institutions.

**Table 2:** Top 10 most productive countries and institutions.

Rank	Country	Publications	Institution	Publications
1	USA	263	Univ Messina	22
2	China	203	Harvard Med Sch	22
3	Italy	137	Med Univ Warsaw	18
4	Turkey	88	Mayo Clin	17
5	UK	81	Univ Turin	15
6	Poland	66	Univ Amsterdam	15
7	India	61	Univ Milan	14
8	Germany	60	Shanghai Jiao Tong Univ	13
9	Netherlands	56	Childrens Hosp Philadelphia	13
10	Japan	50	Erasmus MC	13
	Total (Top 10)	1065	Total (Top 10)	162

### 3.4 Core authors and collaboration networks

**Figure 4:**
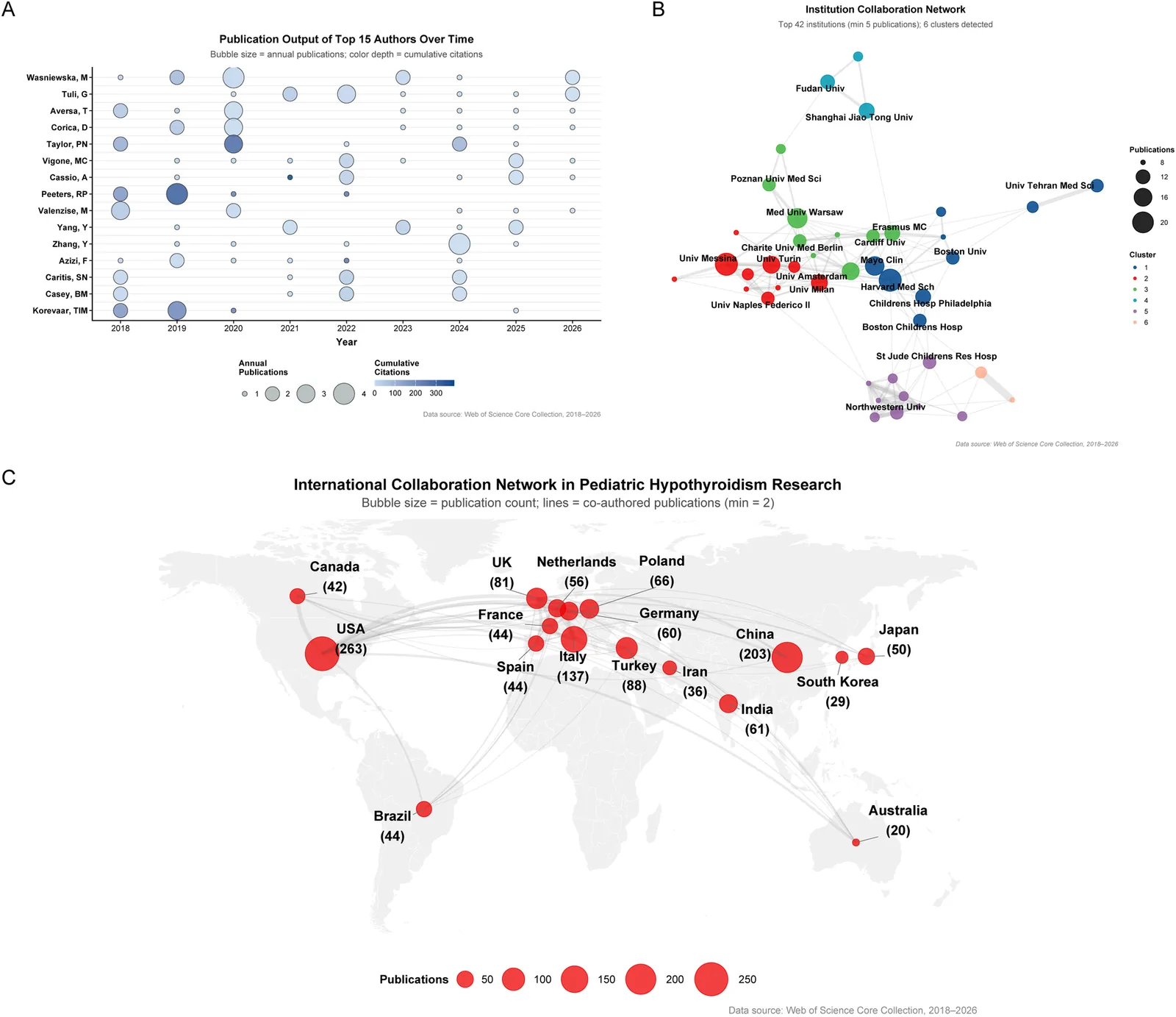
Core authors and multi–level collaboration networks. **(A)** Bubble chart of the top 15 authors’ output and citation impact over time (bubble size = annual publication output; color depth = cumulative citations). **(B)** Institutional collaboration network (node size = publication output; colors = distinct thematic clusters; minimum threshold = 5). **(C)** Geographic visualization of international collaboration (bubble size = national publication output; lines = co-authorship relationships; minimum co-authorshi*p* = 2).

The author productivity analysis identified Wasniewska M (University of Messina, Italy) as the most prolific author with 12 publications, followed by Tuli G (*n* = 11), Aversa T (*n* = 10), and Vigone MC and Taylor PN with 9 publications each (Figure 4A). The majority of the top-15 authors were affiliated with Italian institutions, underscoring Italy's central role in this research domain. International contributors included Taylor PN (United Kingdom), Peeters RP (the Netherlands), and Yang Y and Zhang Y (China).

The institutional collaboration network identified six major collaborative clusters (Figure 4B). The University of Messina and other Italian institutions formed the central collaboration hub, with strong connections to Harvard Medical School, Boston Children's Hospital, Fudan University, and Shanghai Jiao Tong University.

The geographic collaboration network further demonstrated extensive international cooperation (Figure 4C). The United States (*n* = 263) and China (*n* = 203) had the highest publication output and collaboration activity, while Italy (*n* = 137) and the United Kingdom (*n* = 81) served as major European contributors. Table 3 summarizes the publication counts of the most productive authors and journals.

**Table 3:** Top 10 most active journals and authors.

Rank	Journal	Publications	Author	Publications
1	Frontiers in Endocrinology	68	Wasniewska, M	12
2	Journal of Pediatric Endocrinology & Metabolism	60	Tuli, G	11
3	Journal of Clinical Endocrinology & Metabolism	45	Aversa, T	10
4	Hormone Research in Paediatrics	34	Vigone, MC	9
5	Thyroid	28	Taylor, PN	9
6	Children-Basel	23	Corica, D	9
7	Journal of Clinical Research in Pediatric Endocrinology	23	Cassio, A	8
8	Frontiers in Pediatrics	23	Peeters, RP	8
9	BMC Pediatrics	20	Valenzise, M	8
10	Journal of Endocrinological Investigation	19	Yang, Y	8

### 3.5 Journal distribution

**Figure 5:**
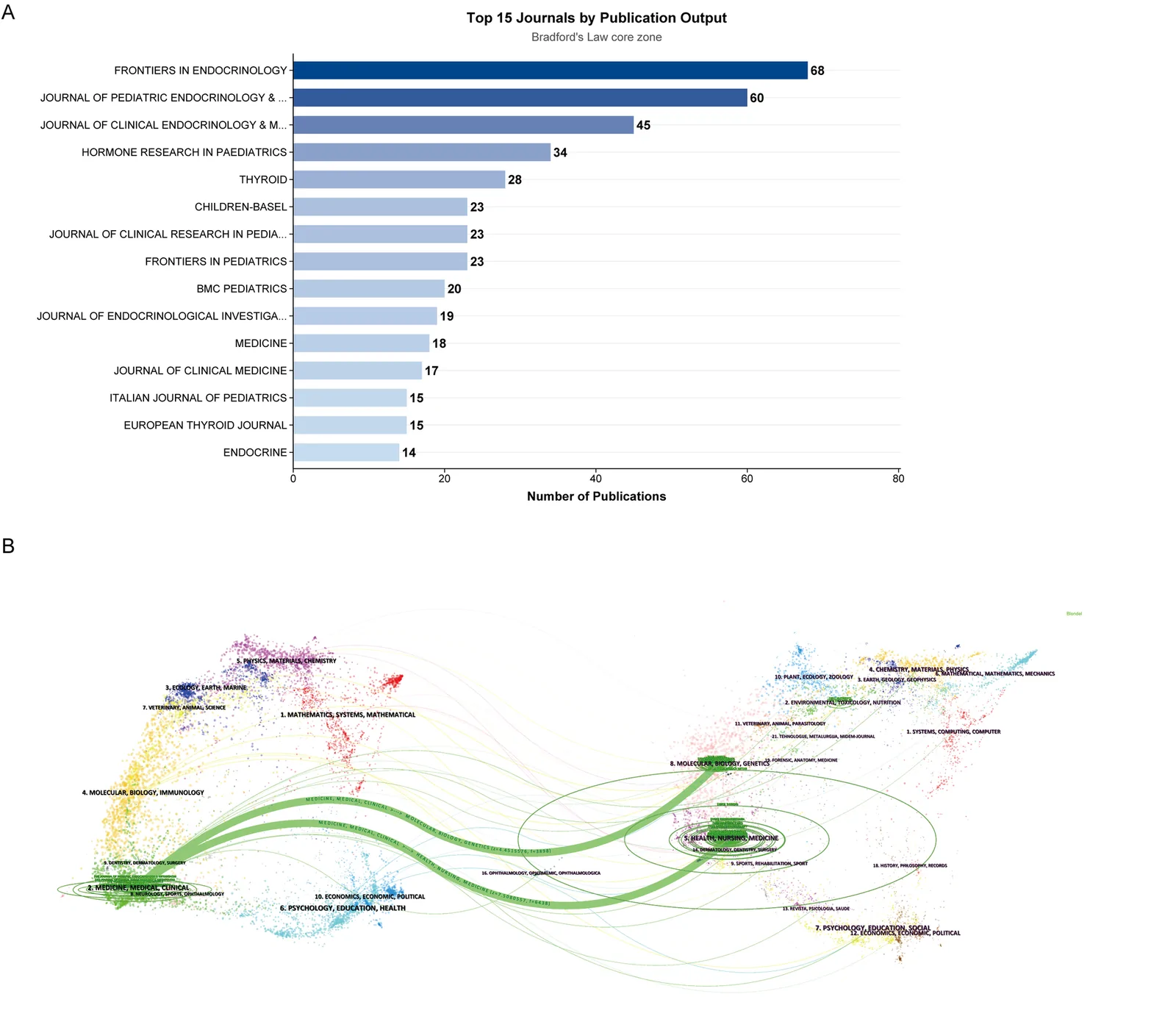
Journal productivity and citation trajectories. **(A)** The top 15 most productive journals publishing research on pediatric hypothyroidism treatment. **(B)** Dual-map overlay of journals. The citing journals on the left represent the research frontiers, while the cited journals on the right represent the intellectual bases. The colored paths indicate the primary citation trajectories between the disciplinary domains.

From 2018 to 2026, a total of 454 journals published articles related to pediatric hypothyroidism treatment. As shown in Figure 5A, Frontiers in Endocrinology was the most productive journal with 68 publications (5.2%), followed by Journal of Pediatric Endocrinology & Metabolism (*n* = 60, 4.6%) and Journal of Clinical Endocrinology & Metabolism (*n* = 45, 3.5%). The top 10 journals collectively accounted for 340 publications (26.1%). The distribution of publications across journals was consistent with Bradford's Law, with a core zone of approximately 10 journals contributing to roughly one-quarter of the total output. Notably, the presence of Thyroid (*n* = 28) and Hormone Research in Paediatrics (*n* = 34) among the top journals reflects the intersection of thyroidology and pediatric endocrinology as the primary disciplinary focus.

Furthermore, a dual-map overlay analysis was conducted to visualize the citation trajectories and map the flow of knowledge at the disciplinary level (Figure 5B). In this overlay, the citing journals on the left represent the current research frontiers, while the cited journals on the right constitute the intellectual bases. The analysis revealed two primary citation pathways. The dominant trajectory originated from journals in the “Medicine, Medical, Clinical” domain and pointed toward cited journals in the “Molecular, Biology, Genetics” domain, indicating that current clinical research on pediatric hypothyroidism heavily relies on fundamental molecular and genetic discoveries, such as genetic screening for thyroid dysgenesis (25). A secondary trajectory linked the clinical frontiers to intellectual bases in the “Health, Nursing, Medicine” domain, reflecting the essential role of public health initiatives, such as newborn screening networks and long-term care management in this field (26).

### 3.6 Keyword Co-occurrence and research hotspots

After standardization using a custom thesaurus file to merge case variants, abbreviations, and synonymous terms, the author keyword analysis identified “hypothyroidism” (*n* = 211), “congenital hypothyroidism” (*n* = 189), “pregnancy” (*n* = 87), “children” (*n* = 86), “subclinical hypothyroidism” (*n* = 75), and “levothyroxine” (*n* = 58) as the most frequently occurring terms.

**Figure 6:**
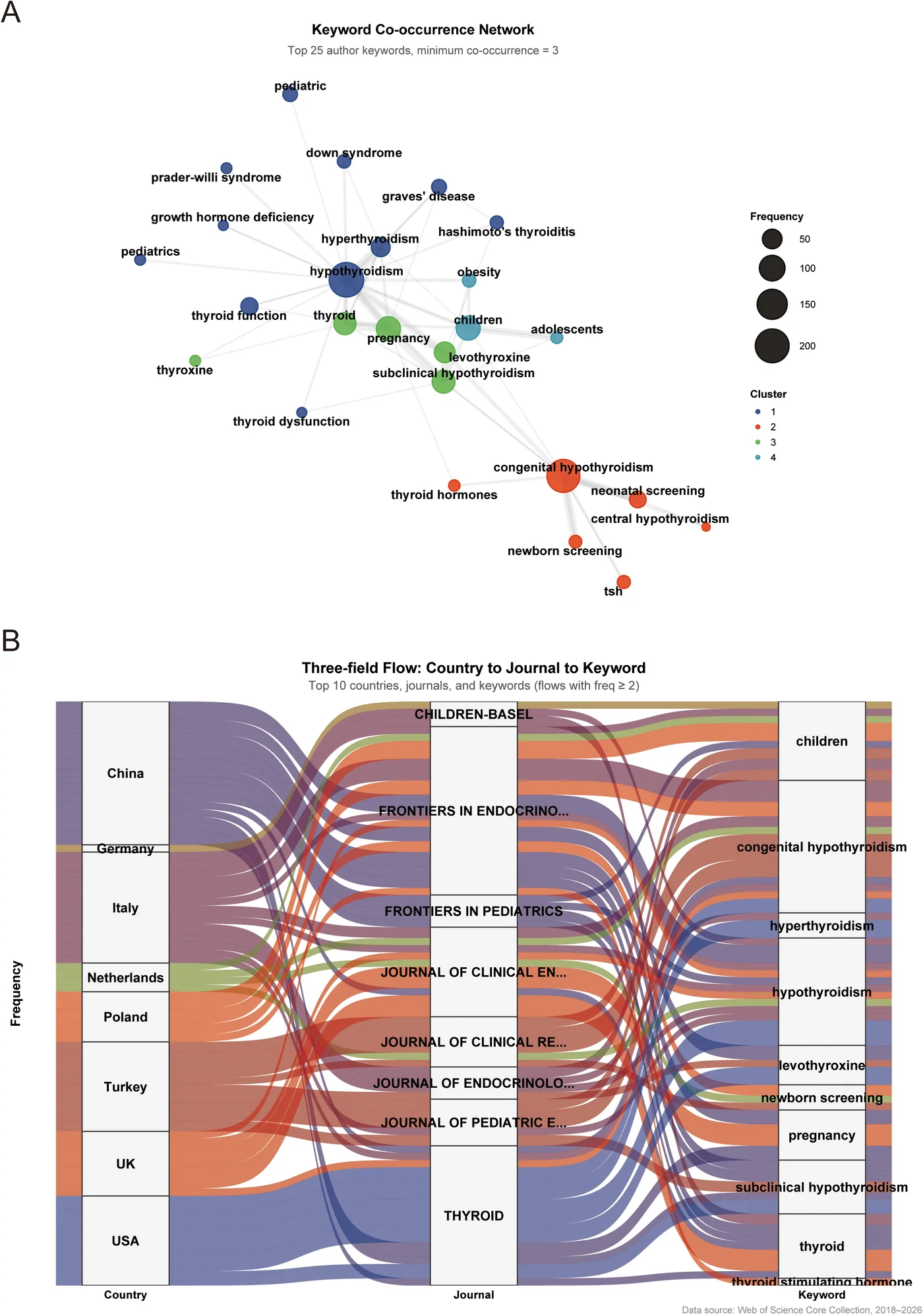
Knowledge structure and multidimensional relationships in pediatric hypothyroidism treatment research. **(A)** Keyword co-occurrence network. Node size is proportional to keyword occurrence frequency, and only keywords with a minimum occurrence of five were included. Different colors represent thematic clusters identified using the Louvain clustering algorithm. **(B)** Three-field Sankey diagram showing the relationships among the most productive countries, journals, and keywords. The width of each flow is proportional to the strength of the relationship.

Keyword co-occurrence analysis combined with Louvain community detection identified five major thematic clusters (Figure 6A**)**

**Cluster 1:** Congenital Hypothyroidism and Neonatal Screening. This cluster included congenital hypothyroidism, newborn screening, levothyroxine, thyroid dysgenesis, dyshormonogenesis, and TSH, reflecting research on early diagnosis and treatment (27).
**Cluster 2:** Subclinical Hypothyroidism and Thyroid Function. This cluster revolved around “subclinical hypothyroidism,” linked to “thyroid function,” “TSH,” “levothyroxine,” and “children.” The strong association between “subclinical hypothyroidism” and “pregnancy” (co-occurrence = 16) reflects growing attention to the impact of mild thyroid dysfunction on obstetric and neonatal outcomes (28, 29).
**Cluster 3:** Pregnancy-Related Thyroid Disorders. The keyword “pregnancy” formed a distinct cluster with links to “thyroid function,” “subclinical hypothyroidism,” “hypothyroxinemia,” and “pregnancy outcomes.” This cluster highlights the expanding research interest in gestational thyroid physiology and its implications for fetal development (30).
**Cluster 4:** Autoimmune Thyroid Disease. This cluster included “Hashimoto's thyroiditis,” “Graves’ disease,” “hyperthyroidism,” and “thyroid autoantibodies,” representing the autoimmune spectrum of thyroid disorders in the pediatric population (31).
**Cluster 5:** Comorbidities and Genetic Syndromes. A smaller but notable cluster linked “obesity,” “Down syndrome,” “growth hormone deficiency,” and “short stature” to thyroid dysfunction, reflecting the multisystem impact of thyroid hormone deficiency in children (32). To intuitively demonstrate the multidimensional relational flow among geographic regions, publication platforms, and core topics, a three-field Sankey diagram was constructed (Figure 6B). This visualization maps how leading countries distribute their research output across core journals and sequentially drive specific thematic keywords within the discipline.
Table 4 summarizes the ten most highly cited publications in this field. Most were international consensus guidelines and high-impact reviews focusing on congenital hypothyroidism, maternal thyroid function, and pediatric thyroid disorders.

**Table 4:** Top 10 most cited publications.

Rank	First Author (Year)	Title	Journal	Citations	DOI
1	van Trotsenburg, P (2021) (2)	Congenital Hypothyroidism: A 2020-2021 Consensus Guidelines Update-An ENDO-European Reference Network Initiative Endorsed by the European Society for Pediatric Endocrinology and the European Society for Endocrinology	Thyroid	388	10.1089/thy.2020.0333
2	Chaker, L (2022)	Hypothyroidism	Nature Reviews Disease Primers	253	10.1038/s41572-022-00357-7
3	Derakhshan, A (2020)	Association of maternal thyroid function with birthweight: a systematic review and individual-participant data meta-analysis	Lancet Diabetes & Endocrinology	227	10.1016/S2213-8587 (20)30061-9
4	Williams, GR (2018)	Thyroid diseases and bone health	Journal of Endocrinological Investigation	224	10.1007/s40618-017-0753-4
5	Persani, L (2018)	2018 European Thyroid Association (ETA) Guidelines on the Diagnosis and Management of Central Hypothyroidism	European Thyroid Journal	176	10.1159/000491388
6	Thompson, W (2018)	Maternal thyroid hormone insufficiency during pregnancy and risk of neurodevelopmental disorders in offspring: A systematic review and meta-analysis	Clinical Endocrinology	162	10.1111/cen.13550
7	Jansen, TA (2019)	Maternal thyroid function during pregnancy and child brain morphology: a time window-specific analysis of a prospective cohort	Lancet Diabetes & Endocrinology	157	10.1016/S2213-8587 (19)30153-6
8	Prezioso, G (2018) (4)	Effect of Thyroid Hormones on Neurons and Neurodevelopment	Hormone Research in Paediatrics	144	10.1159/000492129
9	Lee, SY (2020)	Associations Between Maternal Thyroid Function in Pregnancy and Obstetric and Perinatal Outcomes	Journal of Clinical Endocrinology & Metabolism	134	10.1210/clinem/dgz275
10	Wassner, AJ (2018)	Congenital Hypothyroidism	Clinics in Perinatology	132	10.1016/j.clp.2017.10.004

### 3.7 Emerging research fronts

**Figure 7:**
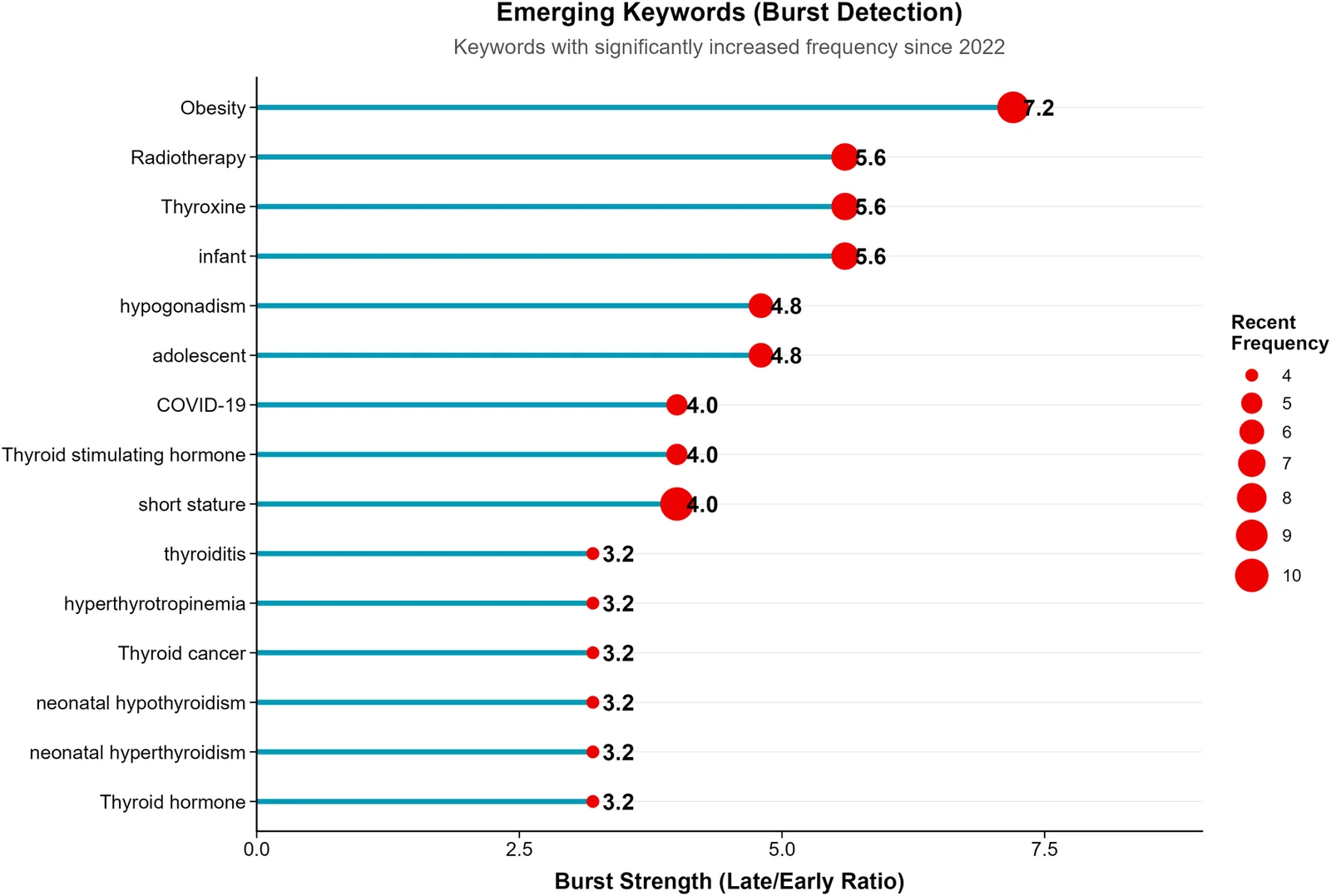
Top 20 keywords with the strongest citation bursts. Gray bars represent the entire study period (2018–2026), whereas red segments indicate the duration of burst activity for each keyword. Numbers adjacent to each keyword represent burst strength.

The burst detection analysis (33) identified 20 keywords with significantly increased frequency in the latter half of the study period (2022–2026), indicating emerging research fronts (Figure 7). The strongest burst was observed for “obesity” (burst strength = 7.2), suggesting increasing recognition of the interrelationship between thyroid dysfunction and pediatric obesity (34). Other notable emerging keywords included “infant” (5.6), “hypogonadism” (4.8), “adolescent” (4.8), “COVID-19” (4.0), “short stature” (4.0), and “levothyroxine treatment” (3.2). The emergence of “COVID-19” as a burst term reflects the recent investigation of SARS-CoV-2 infection and vaccination on thyroid function in children (35). The appearance of “thyroid dyshormonogenesis” (3.2) and “neonatal hypothyroidism” (3.2) suggests renewed interest in the genetic and molecular basis of congenital thyroid disorders (36–38).

## 4 Discussion

### 4.1 Publication trends and geographic landscape

The present bibliometric analysis identified 1,302 publications on pediatric hypothyroidism treatment published between 2018 and 2026, revealing a sustained upward trajectory in research output. The annual publication count increased by approximately 32% from 2018 (*n* = 138) to the peak year of 2022 (*n* = 183), reflecting the growing scientific interest in this field. This growth may be attributed to several converging factors: the expansion of newborn screening programs across developing nations, the introduction of revised international guidelines, and an increasing recognition that mild thyroid dysfunction in children warrants more nuanced management than previously appreciated (39–41).

The United States emerged as the leading contributor, accounting for 263 publications (20.2%), followed by China (*n* = 203, 15.6%) and Italy (*n* = 137, 10.5%). The preeminence of the United States is primarily driven by its extensive network of advanced pediatric endocrine centers and robust funding for long-term clinical cohorts. China's prominent position likely reflects the rapid expansion of its neonatal screening infrastructure, which now covers over 98% of newborns in urban areas, combined with substantial government investment in pediatric endocrinology research (42). Italy's strong representation is consistent with its historically robust tradition in pediatric thyroidology; Italian research groups have produced seminal work on congenital hypothyroidism follow-up, thyroid dysgenesis genetics, and long-term neurodevelopmental outcomes (43). The presence of Turkey, Poland, and the UK among the top contributors further illustrates the global diffusion of pediatric thyroid research.

### 4.2 Intellectual foundations of the field

The intellectual foundation of this field rests on several landmark studies that established the clinical paradigms still in use today. Bongers-Schokking et al. demonstrated in a prospective cohort that the timing and adequacy of L-T4 replacement in neonates with CH directly determine neurodevelopmental trajectories, with earlier initiation and higher initial doses yielding superior cognitive outcomes at school age (44). This study provided the evidentiary basis for the current recommendation of initiating L-T4 within the first two weeks of life. Subsequently, Selva et al. refined this framework by showing that an initial L-T4 dose of 50 μg/day (approximately 10–15 μg/kg/day), rather than lower doses, was associated with significantly higher IQ scores at age 5, reinforcing the principle that prompt and adequate dosing is critical. These foundational studies, together with the ESPE consensus guidelines (45), have collectively shaped the current standard of care: universal neonatal screening, early L-T4 initiation, and TSH-guided dose titration.

### 4.3 Research hotspots in pediatric hypothyroidism treatment

#### 4.3.1 Congenital hypothyroidism and neonatal screening

The keyword co-occurrence analysis identified “congenital hypothyroidism” and “newborn screening” as the most densely connected node pair (co-occurrence = 14), confirming that early detection and treatment of CH remains the central axis of this research field (46). The continued high publication volume on this topic reflects ongoing efforts to optimize screening algorithms, including debates on TSH cutoff thresholds for referral, the role of thyroid imaging in etiological classification, and the long-term neurocognitive follow-up of children identified through screening (47).

#### 4.3.2 Subclinical hypothyroidism: the threshold dilemma

Subclinical hypothyroidism (SCH) in children continues to generate substantial controversy. The 2014 European Thyroid Association (ETA) guidelines on SCH management in pregnancy and children acknowledged the paucity of high-quality pediatric evidence and recommended a conservative approach: observation with repeat thyroid function testing for children with TSH between 4.5 and 10 mIU/L, reserving L-T4 treatment for those with TSH persistently above 10 mIU/L, positive thyroid autoantibodies, goiter, or dyslipidemia (48). This position is further substantiated by longitudinal natural history data from a large pediatric cohort, which demonstrated that the majority of children with mildly elevated TSH spontaneously normalized their values over a 5-year period without pharmacological intervention (49). These findings collectively suggest that the field is moving toward a more conservative, watchful-waiting strategy for pediatric SCH, in contrast to the more interventionist approach that prevailed in the early 2000s (50).

#### 4.3.3 Emerging frontiers: the obesity paradox and overuse of levothyroxine

The most striking finding of our burst detection analysis was the emergence of “obesity” as the strongest burst keyword (burst strength = 7.2), reflecting growing research attention in how clinicians and researchers conceptualize the relationship between thyroid function and body weight in children (51). This observation is consistent with accumulating pediatric evidence suggesting that mild TSH elevation in obese children is frequently an adaptive physiological response rather than true primary hypothyroidism.

The pathophysiological mechanism has been elucidated in detail. In obese children, excess adipose tissue produces elevated levels of leptin, which acts on thyrotropin-releasing hormone (TRH) neurons in the paraventricular nucleus of the hypothalamus, stimulating TRH secretion and consequently increasing pituitary TSH output (52, 53). This leptin-mediated activation of the hypothalamic-pituitary-thyroid (HPT) axis results in a mild-to-moderate TSH elevation—typically in the range of 4.5 to 10 mIU/L—with normal or slightly elevated triiodothyronine (T3) levels. Importantly, this hormonal pattern appears to be an adaptive mechanism aimed at increasing resting energy expenditure and facilitating thermogenesis, rather than a pathological state requiring thyroid hormone replacement (53). The observation that TSH and T3 levels consistently normalize following substantial weight loss, without any thyroid medication, provides compelling evidence that these changes are consequences rather than causes of obesity (54).

The clinical implications of this mechanistic understanding are profound. Current pediatric evidence suggests that routine levothyroxine treatment is generally not recommended for obese children with isolated mild TSH elevation in the absence of biochemical or clinical evidence of primary hypothyroidism (55, 56). Lifestyle modification with weight reduction is considered the first-line management strategy for obese children with isolated mild TSH elevation, as thyroid function often normalizes following weight loss (57). Levothyroxine therapy may be considered only in selected children with persistent TSH elevation, positive thyroid autoantibodies, or other clinical evidence suggestive of true hypothyroidism, consistent with current pediatric recommendations and international consensus guidelines (2, 56, 58, 59). Unnecessary levothyroxine treatment may expose children to avoidable adverse effects, including iatrogenic hyperthyroidism, highlighting the importance of individualized treatment decisions and careful biochemical monitoring (55, 60, 61).

#### 4.3.4 COVID-19 and pediatric thyroid dysfunction

The COVID-19 pandemic has introduced a distinct but important dimension to pediatric thyroid research. Our bibliometric analysis identified “COVID-19” as an emerging burst keyword (burst strength = 4.0), reflecting the rapid accumulation of studies investigating the impact of SARS-CoV-2 infection on thyroid function. McCowan et al. reported that the pandemic altered the clinical presentation of thyroid disease in children, with diagnostic delays attributable to reduced access to routine endocrine follow-up during lockdown periods (62). Furthermore, Herczeg et al. conducted a prospective multi-center study demonstrating that SARS-CoV-2 infection can transiently disrupt thyroid function in children, manifesting as non-thyroidal illness syndrome or subacute thyroiditis, although most alterations resolved spontaneously without long-term sequelae (63). These findings highlight the importance of post-pandemic surveillance of thyroid function in children who experienced COVID-19, particularly those with pre-existing thyroid conditions (64). Although the acute phase of the COVID-19 pandemic has ended, the disruptions it caused to healthcare delivery, newborn screening programs, and long-term endocrine follow-up may have lasting implications for pediatric thyroid care. Future studies should determine whether these disruptions have affected the timing of diagnosis, treatment initiation, and long-term outcomes of pediatric hypothyroidism, and should explore strategies to enhance the resilience of pediatric endocrine services against future public health emergencies (2, 64–66).

## 5 Limitations

The present study has several limitations that should be acknowledged. Firstly, while PubMed was successfully utilized for the external validation of temporal trends and thematic concordance, the detailed bibliometric network mapping (such as co-citation and structural coupling analyses) was conducted exclusively on WoSCC metadata due to the specific data-formatting requirements of the visualization software. Secondly, the inclusion of only English language publications may have resulted in a less comprehensive search, potentially underrepresenting contributions from non-Anglophone countries. Thirdly, bibliometric indicators are subject to temporal bias; some recently published high-quality studies (2024–2026) may not have received widespread attention due to their low citation frequency. Fourthly, keyword co-occurrence analysis is sensitive to author terminology choices, and the absence of a standardized keyword ontology in this field may have introduced some heterogeneity. Fifthly, the keyword co-occurrence network comprised 25 nodes and 38 edges, which is relatively small; although Louvain community detection produced interpretable clusters (modularity Q = 0.42), the robustness of the five-cluster structure should ideally be validated with larger datasets or alternative clustering algorithms. Finally, the inclusion of pregnancy-related thyroid studies (*n* = 87 with “pregnancy” as a keyword) may introduce population heterogeneity, as these studies primarily address maternal rather than directly pediatric thyroid physiology. Nevertheless, this study will facilitate researchers in comprehending the pivotal areas and frontiers of pediatric hypothyroidism treatment.

## 6 Conclusions

This bibliometric analysis of 1,302 publications reveals that pediatric hypothyroidism treatment research is undergoing a thematic shift. The field's center of gravity is moving from congenital hypothyroidism screening optimization toward two emerging priorities: standardizing watchful-waiting strategies for mild subclinical hypothyroidism, and differentiating obesity-related TSH elevation from true primary hypothyroidism to avoid levothyroxine over-prescription. Prospective trials addressing these two clinical questions should be prioritized in the coming decade.

## Data Availability

The original contributions presented in the study are included in the article/Supplementary Material, further inquiries can be directed to the corresponding author.
