# Supplementary material for: Bibliometric analysis of research on pediatric hypothyroidism treatment: a global perspective on trends and emerging fronts (2018–2026)
Source: Front Pediatr. 2026 Aug 17;14:1908940. doi: 10.3389/fped.2026.1908940 (PMC13526919; doi:10.3389/fped.2026.1908940)
Supplement: Supplementary file 1 [file Supplementaryfile1.docx]

Supplementary Material

# 1 Supplementary Figures and Tables

## 1.1 Supplementary Figures

Supplementary Figure S1. Cross-database validation of annual publication trends between the WoSCC and PubMed datasets. Spearman correlation analysis revealed a strong positive correlation (Spearman's ρ = 0.90, P = 0.002).

**Table S1.** Cross-database validation of core research hotspots (WoSCC vs. PubMed)

| **Core Thematic Concept** | **WoSCC Keyword Presence** | **PubMed Corresponding Term**  **(MeSH / Author Keyword)** | **PubMed Frequency** | **Thematic**  **Concordance** |
| --- | --- | --- | --- | --- |
| Primary Disease | Congenital hypothyroidism | Congenital hypothyroidism | 469 (OT), 161 (MeSH) | High |
|  | Hypothyroidism | Hypothyroidism | 425 (OT), 245 (MeSH) | High |
|  | Subclinical hypothyroidism | Subclinical hypothyroidism | 113 (OT) | High |
|  | Autoimmune thyroiditis | Hashimoto thyroiditis* | 110 (OT), 96 (MeSH) | High |
| Intervention/Screening | Levothyroxine | Levothyroxine | 375 (MeSH), 129 (OT) | High |
|  | Newborn screening | Newborn screening | 268 (MeSH), 210 (OT) | High |
| Emerging Fronts | Obesity | Obesity | 70 (OT) | High |
|  | COVID-19 | COVID-19 | 42 (OT), 30 (MeSH) | High |
| Diagnostic Marker | TSH | TSH / Thyroid-stimulating hormone | 418 (MeSH), 115 (OT) | High |
| Target Population | Children / Infants | Children / Infants /  Newborn infant | > 2,000 (Combined) | High |

* In the PubMed database, 'autoimmune thyroiditis' is predominantly indexed under the specific MeSH term and author keyword 'Hashimoto thyroiditis', representing identical clinical concepts.

Abbreviations: WoSCC, Web of Science Core Collection; OT, Author Keywords (Other Terms); MeSH, Medical Subject Headings; TSH, thyroid-stimulating hormone.
